# Supplementary figures and images for: Quantitative expression of estrogen, progesterone and human epidermal growth factor receptor-2 and their correlation with immunohistochemistry in breast cancer at Uganda Cancer Institute
Source: PLoS One. 2025 Jan 3;20(1):e0311185. doi: 10.1371/journal.pone.0311185 (PMC11698450; doi:10.1371/journal.pone.0311185)

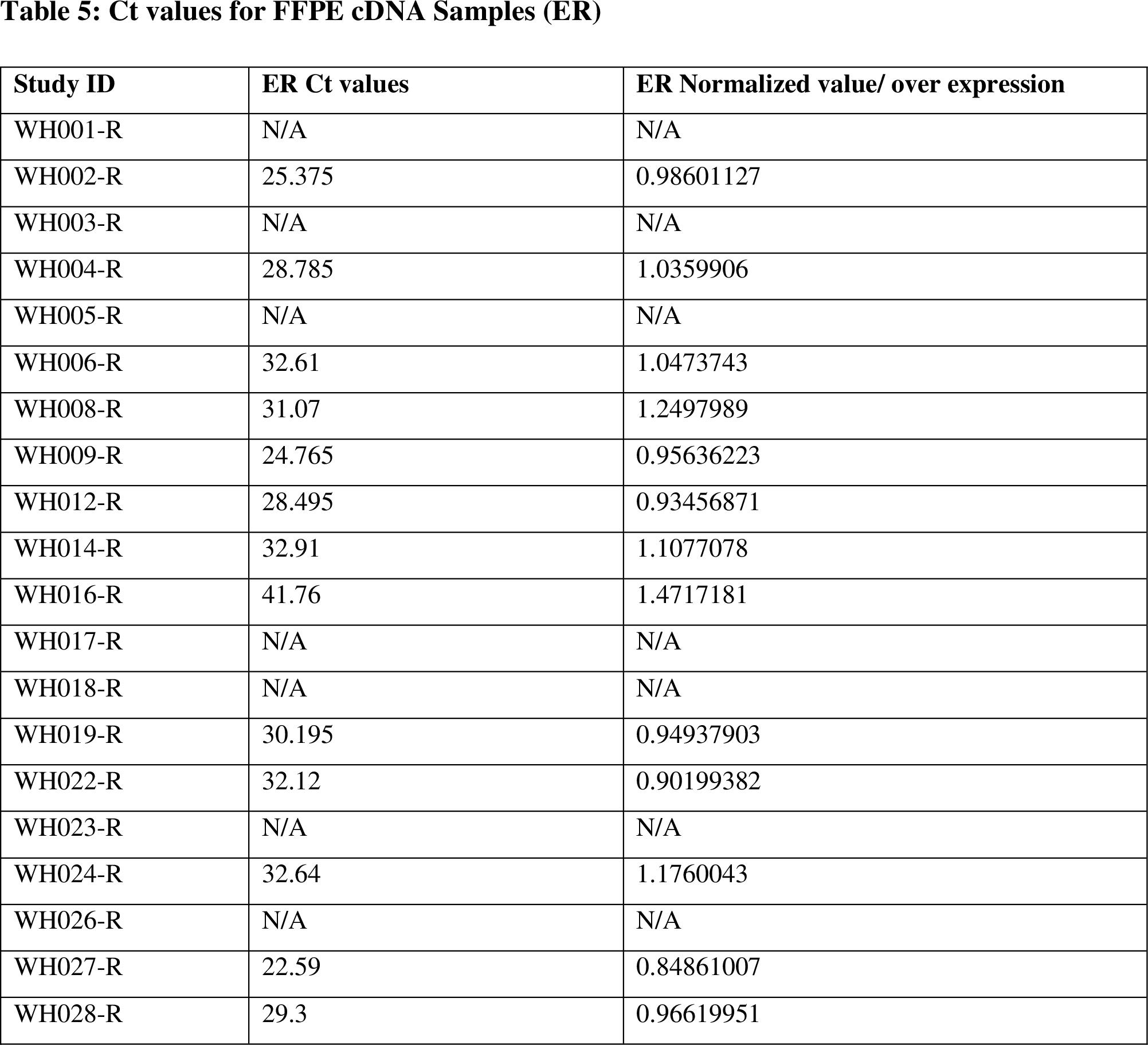

Supplement: S1 Table — (TIF) [file pone.0311185.s002.tif]

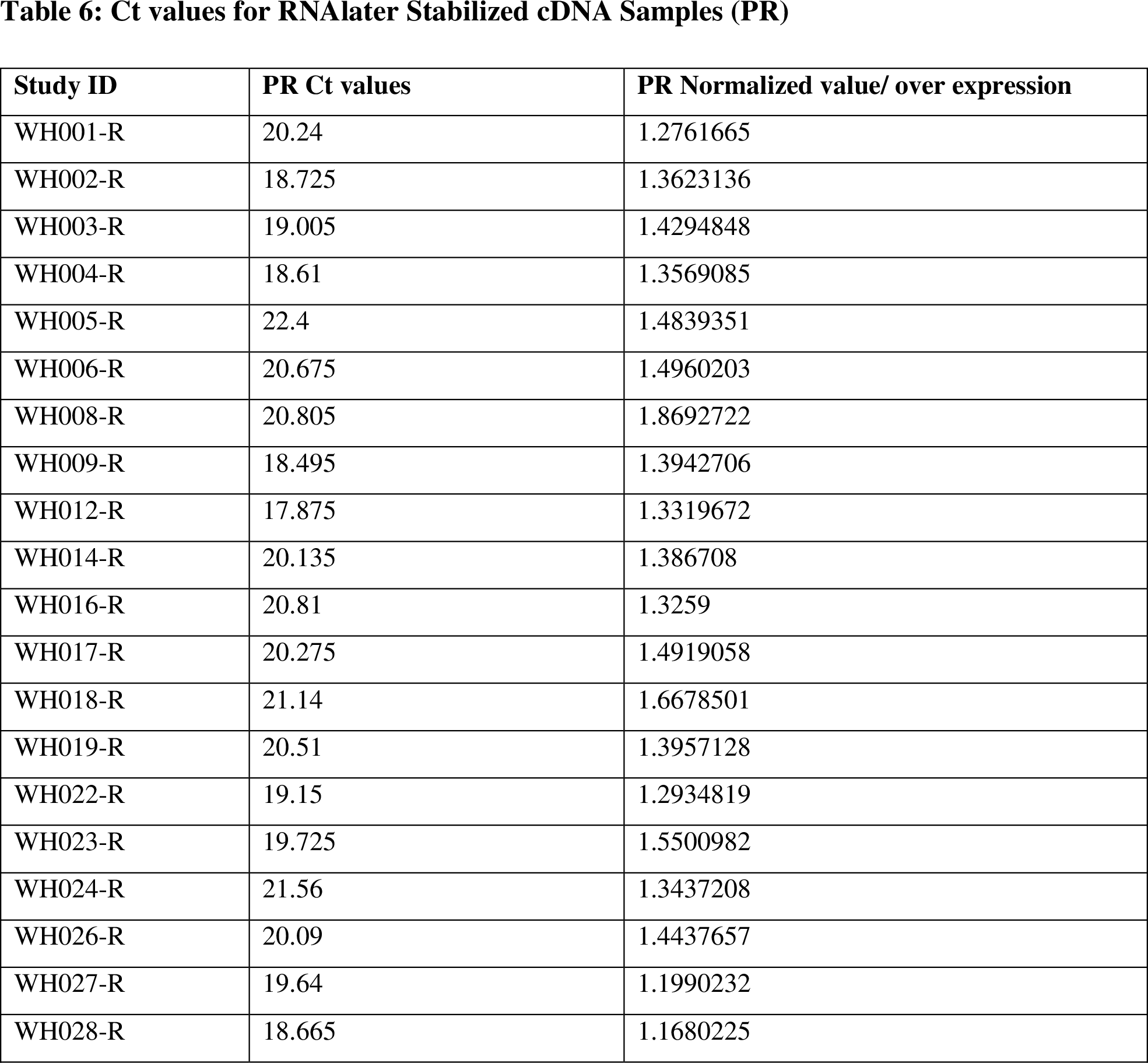

Supplement: S2 Table — (TIF) [file pone.0311185.s003.tif]

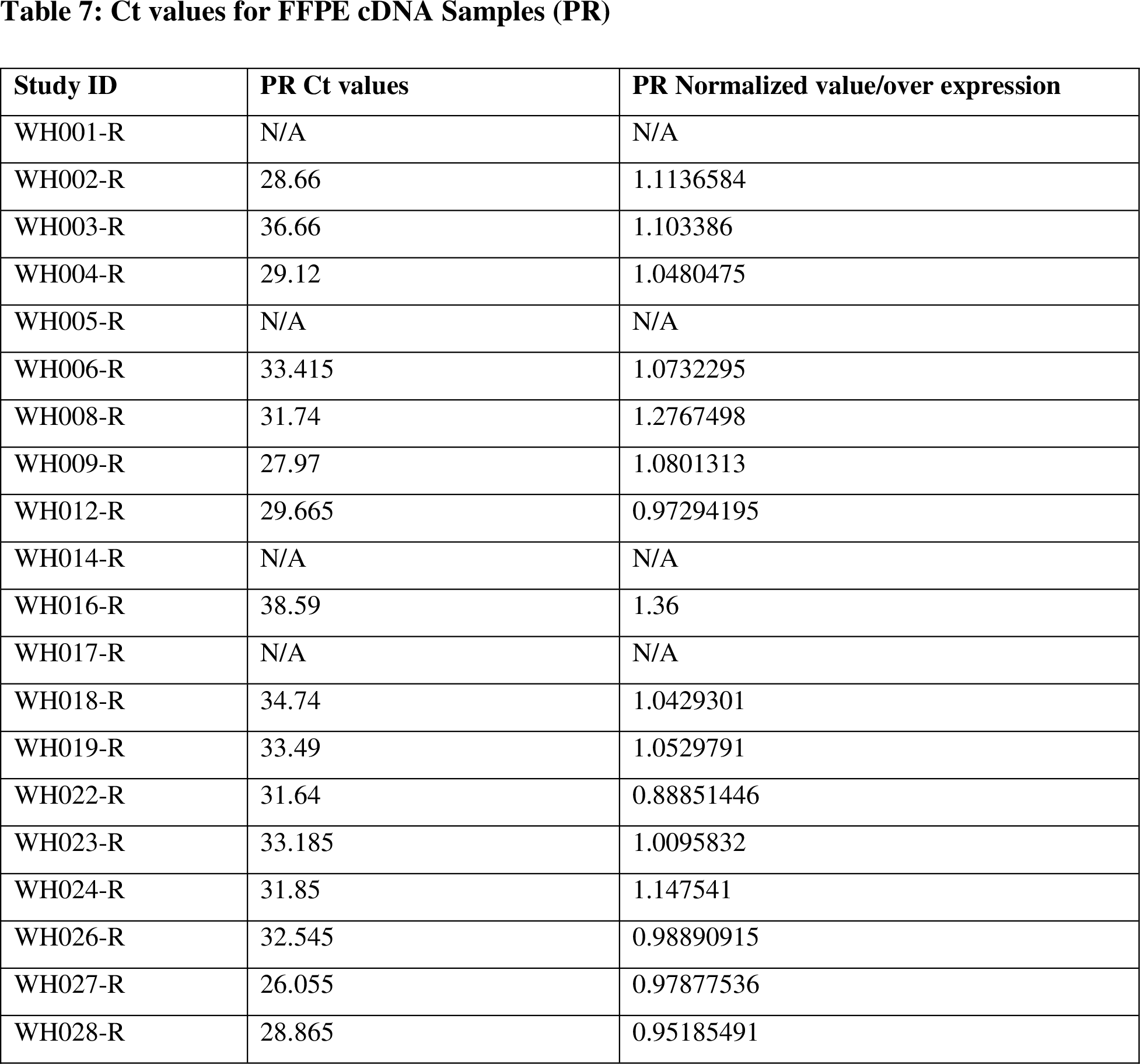

Supplement: S3 Table — (TIF) [file pone.0311185.s004.tif]

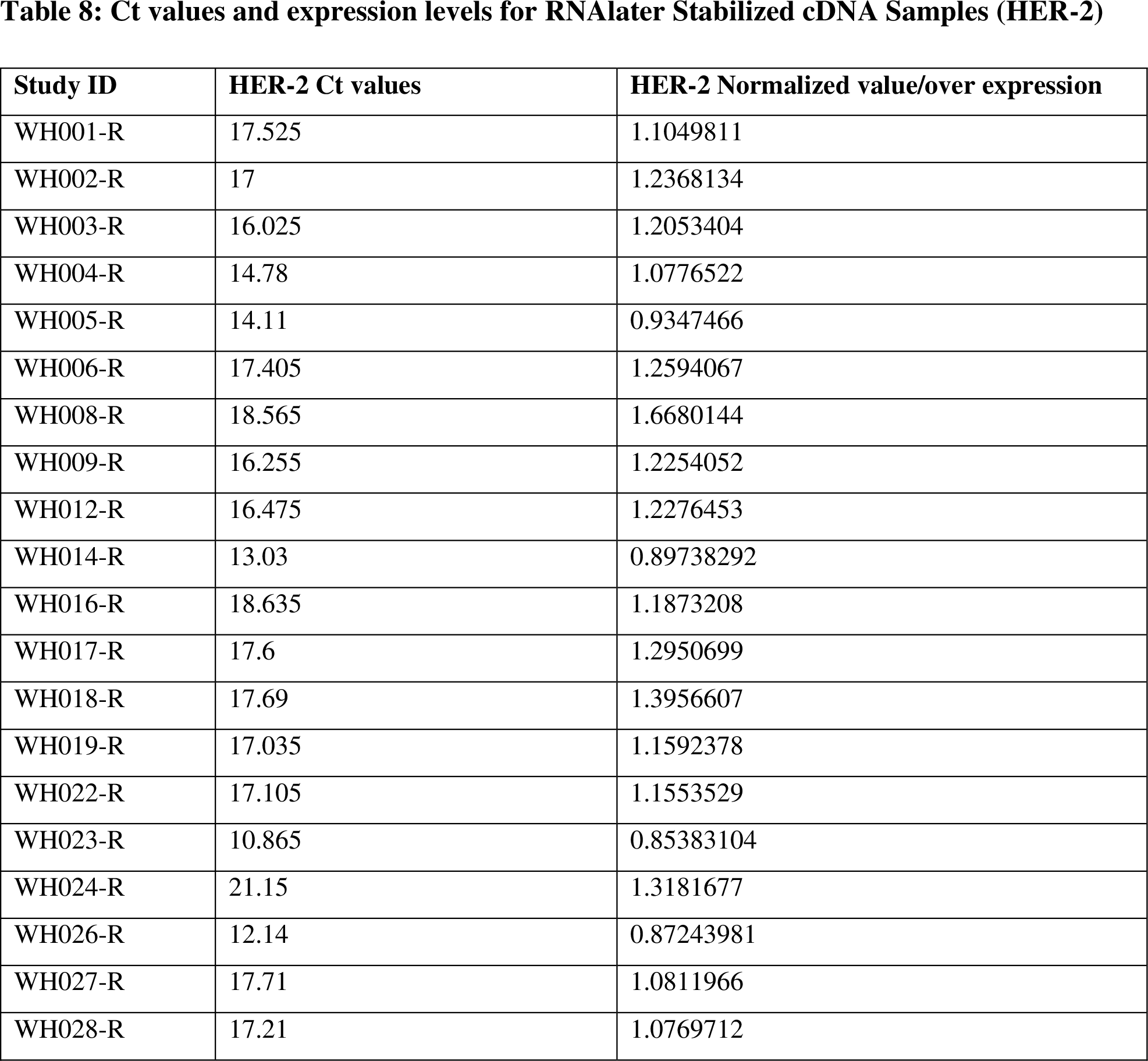

Supplement: S4 Table — (TIF) [file pone.0311185.s005.tif]

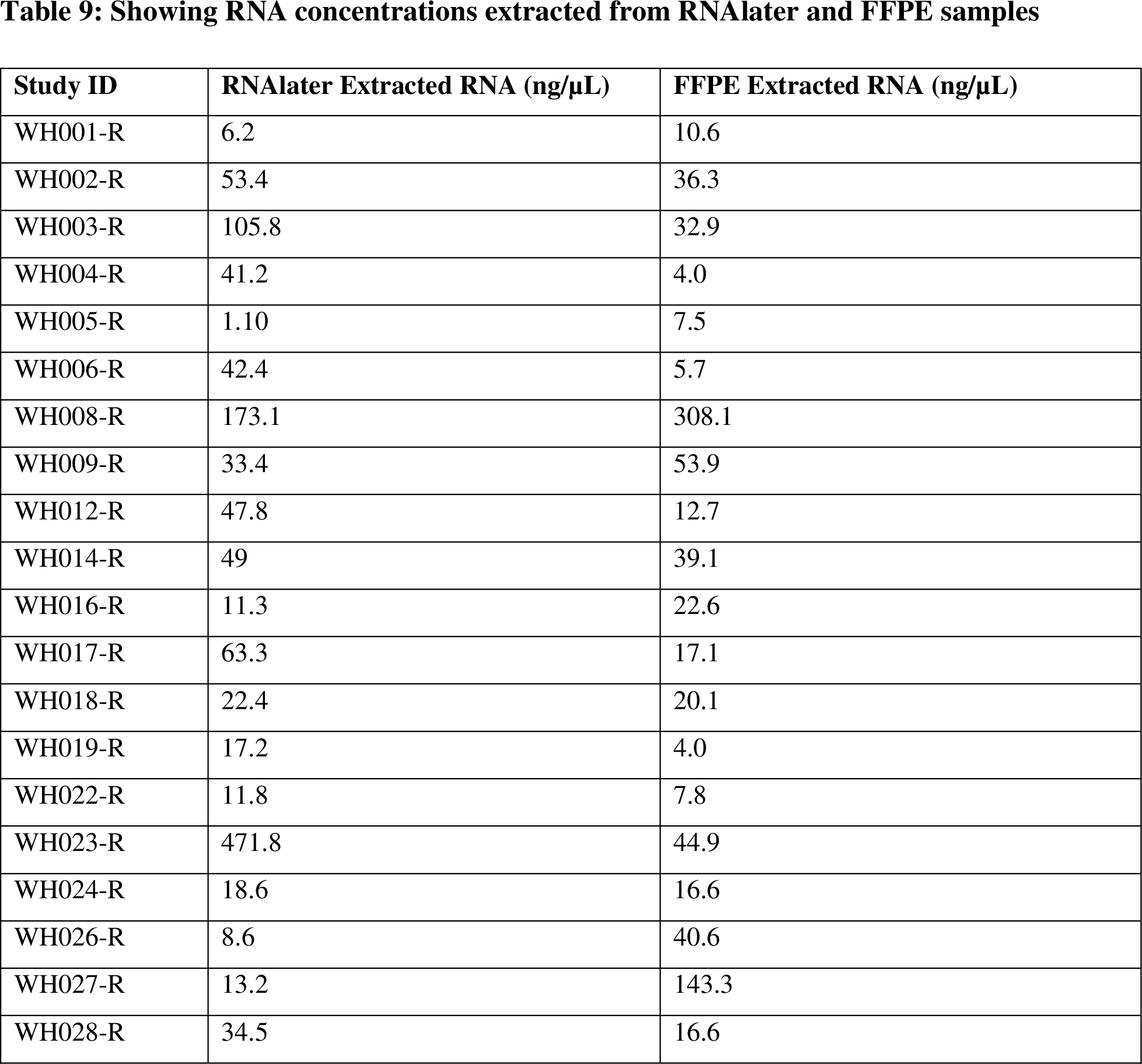

Supplement: S5 Table — (TIF) [file pone.0311185.s006.tif]
