# Supplementary material for: Quantitative expression of estrogen, progesterone and human epidermal growth factor receptor-2 and their correlation with immunohistochemistry in breast cancer at Uganda Cancer Institute
Source: PLoS One. 2025 Jan 3;20(1):e0311185. doi: 10.1371/journal.pone.0311185 (PMC11698450; doi:10.1371/journal.pone.0311185)
Supplement: Attachment — Submitted filename: Response to Reviewers.docx [file pone.0311185.s007.docx]

**11^TH^ AUGUST 2024**

**EDITORIAL BOARD**

**PLOS ONE**

Dear Editor,

**Re: Revised Manuscript: “Quantitative Expression of Estrogen, Progesterone and Human epidermal growth factor receptor-2 and their correlation with Immunohistochemistry in Breast Cancer at Uganda Cancer Institute”**

Thank you for the critical review of our manuscript and the positive suggestions made towards this article to meet the journal requirements.

We have revised the manuscript and on the next page is a table showing the point by point response to each of the comments raised by the reviewers.

Sincerely,

WANNUME HENRY

**RESPONSES TO ALL THE COMMENTS AND ADDITIONS REQUIREMENTS RAISED BY THE REVIEWERS AND EDITOR BOARD.**

| **Sno.** | **COMMENT** | **Correction/Response** | **Page No. (Revised)** |
| --- | --- | --- | --- |
|  | **Reviewer 1** | |  |
| 1 | The study correlates IHC and qPCR data in FFPE samples of breast cancer. The sample size of the study is small. Further, there are subgroups within the limited number of samples like 1+, 2+ and 3+. The argument that RT-PCR is cheaper than IHC seems unjustified. | Sample size was calculated using a formula for minimum sample size calculation and this has been updated on pages 5-6 of the revised manuscript with track changes and in the manuscript. Also with a fact this was a comparison study to identify if quantitative PCR can be done to correctly compare results outcome with immunohistochemistry, 20 samples were considered to be enough for this study. The subgroups of 1+, 2+ and 3+ is an indication of the staining intensity of the tumour cells with the IHC staining antibodies which is the basis of indicating the staining intensity in the IHC stained slides. This positivity level was also correlated with the expression levels under qPCR as a measure of positivity levels just in case a semi quantitative scale is employed. Explanation relating how expensive qPCR compared to IHC is given in the discussion section. However, cost may also be attached to the lots of man power used, the long waiting hours due to the several steps that are involved processing individual tissue samples through grossing to the final steps of IHC staining while qPCR several samples can be processed in one run and final results recorded | Pages: 5 -6 lines; 143-160 |
|  | **Reviewer** |  |  |
| 2 | Authors have stated that data is fully available without restriction, but under the section describing where the data may be found there is a link to the author’s ORCiD account, not a data repository. Kindly correct this and add a link to the data or upload as a separate file. | This has been noted and data has also be attached as a supplementary data file. | Page  Attached x |
| 3 | Please check if format of citations and modify according to journal criteria where needed. | This is noted and the manuscript has been formatted according to journal criteria | Pages;  Entire document. |
| 4 | In Introduction, line number 77, please clarify the following “This is almost three times what is reported in other regions”. | With the reported prevalence of Triple Negative Breast Cancer in Uganda and at the Uganda Cancer institute at 34%, this percentage is almost three times to the levels of TNBC being reported in different countries around the world at 12 – 17% and 5-10%, as reported by Galukande et al, 2024 and Alexandra et al, 2023 respectively. | Page:3 lines; 80-90 |
| 5 | Minor English language proofreading by the authors will improve the manuscript further. | Replaced tumor with tumour in the entire manuscript | Pages; Entire document |
| 6 | The authors propose qPCR as a cheaper and faster method of determining hormonal receptors expression in breast cancer, as compared to IHC. However, in their discussion the authors should also mention the benefits associated with IHC which are missed when using qPCR, for example, it cannot be determined WHERE the markers are being expressed, whether in the tumour core, the margins etc., IHC also informs us regarding tissue architecture of tumour etc. Please mention benefits of both techniques for a more comprehensive discussion. | A section has been included in the discussion of the results the comparison of the merits and demerits of IHC when compared with qPCR have been included and discussed in the manuscript | Pages: 22; Lines; 584-600 |
| 7 | It is not mentioned whether IHC antibodies were diluted for use, or these were ready-to-use clones. | Information about the antibodies that were used in IHC staining has been included in the manuscript and they were ready to use antibody clones. | Page: 5, line 137 |
|  | **Reviewer 3** | | |
| 9 | Thank you for your submission. It was interesting read. However, the study sample size of 20 is too small to draw any concrete conclusion and recommendations. Also, the reference are not marked (numbered) in the body of the manuscript as per the references. The manuscript is otherwise well written. | We appreciate the nice comments; please note that minimum sample size for this study was considered to be sufficient based on the study by Sinn *et al.*, 2017 and also based on the formula for sample size correlation (Cummings *et al.*, 2013). References to be marked in the final manuscript. Also with a fact this was a comparison study to identify if quantitative PCR can be done to correctly compare results outcome with immunohistochemistry, 20 samples were considered to be enough for this study. | Page:5 – 6, lines 142-164 |
|  | **Reviewer 4** | | |
| 10 | The manuscript is well-written and presents an original contribution to the field. Your comprehensive methodology is well-detailed and robust, ensuring the reproducibility and reliability of your findings. The results are compelling and well-supported by data. Overall, your research is a significant addition to the existing literature, and I believe it will be of great interest to the specific group | We sincerely appreciate these encouraging comments about our research and write up. Thank you. |  |
|  | **Additional Requirements** |  |  |
| 11 | During your revisions, please note that a simple title correction is required: We request you to please remove the phrase "Original manuscript Title" from the title of your manuscript. Please ensure this is updated in the manuscript file and the online submission information. | The words indicating original "Original manuscript" has been removed and deleted, with the new title arrangement stating as; “Quantitative Expression of Estrogen, Progesterone and Human epidermal growth factor receptor-2 and their correlation with Immunohistochemistry in Breast Cancer at Uganda Cancer Institute” | Page: 1 line 1 |
| 12 | We note that the grant information you provided in the ‘Funding Information’ and ‘Financial Disclosure’ sections do not match. When you resubmit, please ensure that you provide the correct grant numbers for the awards you received for your study in the ‘Funding Information’ section. | This has been noted, corrected, and an updated funding information section is provided | Page 6; |
| 13 | When completing the data availability statement of the submission form, you indicated that you will make your data available on acceptance. We strongly recommend all authors decide on a data sharing plan before acceptance, as the process can be lengthy and hold up publication timelines. Please note that, though access restrictions are acceptable now, your entire data will need to be made freely accessible if your manuscript is accepted for publication. This policy applies to all data except where public deposition would breach compliance with the protocol approved by your research ethics board. If you are unable to adhere to our open data policy, please kindly revise your statement to explain your reasoning and we will seek the editor's input on an exemption. Please be assured that, once you have provided your new statement, the assessment of your exemption will not hold up the peer review process. | This has been addressed during the online completion of the forms and data sharing has been agreed upon by all the authors before acceptance of the manuscript and the data is hence made freely accessible. Also raw data file has been attached for easy access. | online submission and attached separate file |
| 14 | We note that you have referenced (UCI. (2018). Statistical unpublished data Uganda Cancer Institute) which has currently not yet been accepted for publication. Please remove this from your References and amend this to state in the body of your manuscript: (ie “Bewick et al. [Unpublished]”) as detailed online in our guide for authors | This has been updated as recommended | Reference has been removed |
| 15 | PLOS ONE now requires that authors provide the original uncropped and unadjusted images underlying all blot or gel results reported in a submission’s figures or Supporting Information files. This policy and the journal’s other requirements for blot/gel reporting and figure preparation are described in detail at  When you submit your revised manuscript, please ensure that your figures adhere fully to these guidelines and provide the original underlying images for all blot or gel data reported in your submission. See the following link for instructions on providing the original image data:  In your cover letter, please note whether your blot/gel image data are in Supporting Information or posted at a public data repository, provide the repository URL if relevant, and provide specific details as to which raw blot/gel images, if any, are not available. | This has been noted and a gel in the TIFF image format has been provided. | Page: attached separate file. |
| 16 | Please remove your figures from within your manuscript file, leaving only the individual TIFF/EPS image files, uploaded separately. These will be automatically included in the reviewers’ PDF. | All images have been removed from the manuscript as recommended and individual TIFF separate files attached | Images removed from the entire document and attachments provided as advised |
